# Clinical characteristics and prognostic differences between elderly-onset and adult-onset ulcerative colitis: a two-center retrospective cohort study

**DOI:** 10.3389/fmed.2026.1847513

**Published:** 2026-06-02

**Authors:** Hailong Li, Shigang Ding, Jun Li, Qiao Meng, Ranran Zeng, Fang Gu, Xiangchun Lin

**Affiliations:** 1 Department of Gastroenterology, Peking University Third Hospital, Beijing, China; 2 Beijing Key Laboratory for Helicobacter Pylori Infection and Upper Gastrointestinal Diseases (BZ0371), Beijing, China; 3 Department of Gastroenterology, Peking University International Hospital, Beijing, China

**Keywords:** adult-onset ulcerative colitis, clinical features, elderly-onset ulcerative colitis, prognosis, treatment

## Abstract

**Background:**

The incidence of elderly-onset ulcerative colitis (EO-UC) has been gradually increasing. The unique characteristics of clinical manifestations, therapeutic strategies, and prognosis in EO-UC patients compared with adult-onset ulcerative colitis (AO-UC) patients remain unclear.

**Methods:**

We retrospectively analyzed hospitalized ulcerative colitis (UC) patients from March 2010 to June 2024 at Peking University Third Hospital and Peking University International Hospital. We recruited 68 EO-UC patients (age at diagnosis≥60 years) and 136 AO-UC patients (age at diagnosis <60 years, matched ratio = 1:2). Follow-up lasted from the date of UC diagnosis to the earlier of two endpoints: January 31, 2025, or the date of the last clinical visit at our center. Subsequently, we analyzed the differences in clinical manifestations, therapeutic strategies, and prognosis between the two groups.

**Results:**

The male-to-female ratio in the EO-UC group was 1.8:1, and this group had milder clinical manifestations (abdominal pain 58.5% *vs.* 75.0%, *p* = 0.022; mucous bloody stool 50.0% *vs.* 74.3%, *p* < 0.001) and a higher comorbidity burden (*p* < 0.001) than the AO-UC group. The distribution of lesion locations differed significantly between the two groups (*p* = 0.001), with higher proportions of proctitis and left-sided colitis in the EO-UC group (14.7% *vs*. 2.2% and 22.1% *vs*. 18.4%). The use of corticosteroids and biologics before hospitalization was significantly lower in the EO-UC group than in the AO-UC group (13.2% *vs.* 42.6%, *p* < 0.001; 0.0% *vs.* 13.2%, *p* = 0.002). No significant differences were observed between the two groups in terms of concurrent infections, clinical remission rates, relapse patterns, rehospitalization, or complication occurrence. During follow-up, six surgical cases occurred in the adult-onset group, and two in-hospital all-cause mortality cases occurred in the elderly-onset group.

**Conclusion:**

The EO-UC group was predominantly male, and it exhibited higher comorbidity and concomitant medication rates and milder clinical manifestations than the AO-UC group, presenting challenges for clinical diagnosis. 5-Aminosalicylic acid (5-ASA) remains the first-line therapeutic regimen, and the EO-UC group demonstrated lower utilization rates of immunosuppressants and biologics than the AO-UC group, which may be related to distinct treatment considerations in elderly patients, although this did not translate into observable differences in clinical outcomes between the two groups. This study is preliminary and exploratory, and larger prospective studies with adequate sample sizes are needed to validate these findings in the future.

## 1 Introduction

Ulcerative colitis (UC), a subtype of inflammatory bowel disease (IBD) with a higher incidence than Crohn’s disease (CD), is a chronic relapsing intestinal disorder driven by gene–environment interactions leading to an abnormal immune response to the gut microbiome (1, 2). The incidence of UC is increasing annually. UC incidence rates are highest in the third and fourth decades, follow by the seventh and eighth decades (1, 3, 4). A long-term, large-scale prospective cohort study from Veszprem County, Hungary, involving 1,370 UC patients demonstrated a progressive increase in the proportion of elderly-onset UC (EO-UC) patients across three time periods: 1977–1955 (10.1%), 1996–2008 (12.9%), and 2009–2018 (17.2%) (5). Approximately 25%–35% of IBD patients are over 60 years old, including 10%–15% who were diagnosed after the age of 60 years and 20% who are now in that age range but were diagnosed earlier in life (6–8). A large U.S. database encompassing approximately 56 million citizens revealed that from 2005 to 2015, the highest incidence rate occurred among individuals aged ≥60 years (16–22 cases per 100,000 population annually), followed by adults aged 26–59 years (12–20 cases per 100,000 population annually) (9). These findings highlight the increasing global urgency of developing personalized health care strategies and rationally allocating medical resources for EO-UC patients (10).

Whether differences in the age of UC onset affect prognosis remains controversial (3, 11). Studies have shown that patients with moderate-to-severe EO-UC group exhibit higher treatment failure rates, UC-related colectomy, mortality, and severe infection risks than other classes of UC patients (12). Eun Mi Song et al. reported that the 10-year cumulative colectomy rate, ulcerative colitis–related mortality rate, and all-cause mortality rate of EO-UC patients were higher than those of adult-onset UC (AO-UC) patients (13). Komoto et al. (14) study also reported that EO-UC patients had higher disease severity and a greater risk of hospitalization than AO-UC patients. However, the study by Cheddani et al. (15) did not find a difference in cancer risk between the two groups. EO-UC patients were reported to have more comorbidities than AO-UC patients (69.6% *vs.* 14.7%, *p* < 0.001) and diagnostic complexities requiring differentiation from conditions such as ischemic colitis, vasculitis, and drug-induced colitis (16). Furthermore, Mengmeng Zhang et al. reported that EO-UC patients experienced rapid progression and exhibited poor responses to therapeutic drugs (12). Thus, the EO-UC group may demonstrate unique characteristics in terms of natural disease course, therapeutic drug selection, prognosis, and follow-up strategies. Therapeutic regimen for EO-UC patients must be chosen cautiously, considering aging immunity, susceptibility to infection, and a high incidence of malignancies, along with concomitant medication risks from comorbidity management.

This study aimed to summarize the differences in clinical features between EO-UC patients and AO-UC patients, thereby providing empirical evidence to guide the precise selection of therapeutic regimens. Additionally, we compared the clinical prognosis and outcomes between EO-UC and AO-UC patients. These findings can help to optimize treatment strategies and standardize disease management for EO-UC patients.

## 2 Materials and methods

### 2.1 Study subjects

By searching the electronic medical record systems for hospitalized patients diagnosed with ulcerative colitis at Peking University Third Hospital (March 2010 to June 2024) and Peking University International Hospital (June 2018 to June 2024), we identified patients with a confirmed diagnosis of UC, per the ECCO–ESGAR recommendations (17, 18). A clear diagnosis of UC was established based on the integration of clinical (persistent or recurrent bloody diarrhea, with or without abdominal pain, tenesmus, or urgency), endoscopic (continuous mucosal inflammation, characterized by erythema, granularity, friability, loss of vascular pattern, erosions, or ulcerations), and histopathological findings (distorted crypt architecture, basal plasmacytosis, increased lamina propria inflammatory cell infiltrate, and cryptitis or crypt abscesses). The exclusion criteria were the presence of granulomas or other features suggestive of Crohn’s disease or other intestinal current infection, such as Cytomegalovirus, Epstein–Barr virus, *Mycobacterium tuberculosis*, and so on. EO-UC was defined as a diagnosis that was established at ≥60 years of age, whereas AO-UC was defined as a diagnosis age <60 years. EO-UC and AO-UC patients were matched at a 1:2 ratio on the basis of sex and hospitalization date ±1 year. The exclusion criteria were as follows: (I) age <16 years at admission diagnosis; (II) incomplete medical records; and (III) no clear diagnosis of UC. Follow-up was conducted for EO-UC and AO-UC patients. All patients were followed from the time of UC diagnosis to the earlier of two endpoints: January 31, 2025, or the last clinical visit at our center. Patients lost to follow-up were contacted via telephone for follow-up. The primary mortality endpoint was in-hospital all-cause mortality. Death events were identified through electronic medical records and confirmed by reviewing discharge summaries and death certificates when available.

### 2.2 Demographic and clinical characteristics of UC patients

Demographic and clinical data including sex, age at consultation/diagnosis, length of hospital stay, comorbidities and concomitant medications, smoking/alcohol history, clinical symptoms, laboratory tests, complications, concurrent infections, and therapeutic regimens were collected. Follow-up was conducted until the latest available medical record or January 31, 2025, whichever occurred first, and the following outcomes were monitored: clinical remission, recurrence, rehospitalization, complications, infections, UC-related colectomy, in-hospital all-cause mortality, and therapeutic regimen adjustments. The Charlson Comorbidity Index (CCI) was used to assess the burden of comorbidities by assigning weighted scores to 17–19 underlying diseases. Concomitant medication was defined as the concurrent use of one or more chronic non-UC prescription medications. UC clinical phenotypes were classified as initial onset and chronic relapsing. Initial-onset UC referred to the first episode without a prior history of disease, whereas chronic relapsing UC was defined as symptom recurrence during clinical remission. Disease activity was evaluated using the modified Mayo score: mild activity (3–5 points), moderate activity (6–10 points), and severe activity (11–12 points). Clinical remission was defined as a modified Mayo score < 2 points with no individual subscore > 1 point. The main infections recorded as complications were Epstein–Barr virus (EBV), cytomegalovirus (CMV), *Clostridioides difficile*, and fungal infections.

### 2.3 Statistical methods

Data analysis was conducted using SPSS 29.0 (SPSS, Chicago, IL, USA) and GraphPad Prism (GraphPad Software, San Diego, CA, USA). Continuous variables are expressed as the mean ± standard deviation or median (interquartile range) as appropriate. Categorical data are expressed as frequencies (percentages). The data were compared using t tests for normally distributed continuous variables, Wilcoxon–Mann–Whitney tests for nonnormally distributed variables, and chi-square tests or Fisher’s exact tests for noncontinuous variables. Kaplan–Meier analysis was used to assess the cumulative probabilities of infection and complication occurrence, surgical interventions, and in-hospital all-cause mortality. A *p*-value < 0.05 was taken to indicate statistical significance.

## 3 Results

### 3.1 Baseline characteristics

A total of 68 UC patients who were diagnosed with UC at ≥60 years of age were included, and a matched cohort of 136 patients with AO-UC (aged <60 years) was established, with a comparable sex distribution and similar hospital admission dates. We first analyzed the baseline clinical characteristics of the UC patients. There were significant intergroup differences in age at consultation [67.00(64.00, 72.75)*vs*. 34.00(27.00, 43.75)years, *p* < 0.001], age at diagnosis [64.00(62.00, 68.00)*vs*. 30.00(24.00, 38.00)years, *p* < 0.001], duration of hospitalization (16.43 ± 11.19 *vs*. 13.31 ± 9.25 days, *p* = 0.036), body mass index [23.63(20.55, 25.83)*vs.* 20.76(18.78, 23.80)kg/m^2^, *p* = 0.002], comorbidities (hyperlipidemia 17.6% *vs*. 2.2%, *p* < 0.001; hypertension 35.3% *vs*. 3.7%, *p* < 0.001; diabetes mellitus 25.0% *vs*. 2.9%, *p* < 0.001; coronary heart disease 10.3% *vs*. 0.0%, *p* < 0.001; cerebrovascular disease 17.6% *vs*. 0.7%, *p* < 0.001), and Charlson Comorbidity Index (*p* < 0.001), concomitant medication (27.9% *vs*. 5.9%, *p* < 0.001), smoking and Alcohol consumption. Detailed information is provided in Table 1.

**Table 1:** Baseline characteristics.

Baseline characteristics	EO-UC (*n* = 68)	AO-UC (*n* = 136)	*p*
Sex (male)	44 (64.7%)	88 (64.7%)	1.0
Age of consultation (years)	67.00 (64.00, 72.75)	34.00 (27.00, 43.75)	<0.001
Disease course (years)	3.82 ± 4.09	4.43 ± 5.20	0.394
Age at diagnosis (years)	64.00 (62.00, 68.00)	30.00 (24.00, 38.00)	<0.001
Duration of hospitalization (days)	16.43 ± 11.19	13.31 ± 9.25	0.036
BMI (kg/m^2^)	23.63 (20.55, 25.83)	20.76 (18.78, 23.80)	0.002
Comorbidity			
Hyperlipidemia	12 (17.6%)	3 (2.2%)	<0.001
Hypertension	24 (35.3%)	5 (3.7%)	<0.001
Diabetes mellitus	17 (25.0%)	4 (2.9%)	<0.001
Coronary heart disease	7 (10.3%)	0 (0.0%)	<0.001
Cerebrovascular disease	12 (17.6%)	1 (0.7%)	<0.001
Autoimmune disease	3 (4.4%)	9 (6.6%)	0.754
Charlson comorbidity index			<0.001
0	24 (35.3%)	122 (89.7%)	
1	25 (36.8%)	9 (6.6%)	
2	16 (23.5%)	5 (3.7%)	
3	3 (4.4%)	0 (0.0%)	
Concomitant medication (≥1 type)	19 (27.9%)	8 (5.9%)	<0.001
Smoking			<0.001
Nonsmoking patients	32 (47.1%)	112 (82.4%)	
Former smokers	30 (44.1%)	9 (6.6%)	
Current smokers	6 (8.8%)	15 (11.0%)	
Alcohol consumption	22 (32.4%)	25 (18.4%)	0.034
Family history (Gastrointestinal cancer/IBD)	2 (2.9%)	9 (6.6%)	0.343

UC, ulcerative colitis; BMI, body mass index. Data are presented as the mean ± standard deviation, median (interquartile range) or *n* (percentage).

Table 2 presents the clinical manifestations in the UC patients. The rates of abdominal pain (58.5% *vs*. 75.0%, *p* = 0.022), mucous bloody stool (50.0% *vs*. 74.3%, *p* < 0.001), and fever (19.1% *vs*. 39.0%, *p* = 0.007) were lower in the EO-UC group than in the AO-UC group. In addition, the EO-UC group exhibited higher rates of hepatobiliary diseases (19.1% *vs*. 6.6%, *p* = 0.009). No significant differences were observed in diarrhea, tenesmus, articular involvement (arthritis of the shoulder, wrist, knee, and ankle; ankylosing spondylitis; and other related conditions), mucocutaneous manifestations (oral ulcers, erythema nodosum, pyoderma gangrenosum, etc.), ocular lesions (iritis, scleritis, uveitis, etc.) or thromboembolic diseases (arterial and venous thrombosis).

**Table 2:** Clinical manifestations.

Symptoms	EO-UC (*n* = 68)	AO-UC (*n* = 136)	*p*
Intestinal manifestations			
Abdominal pain	38 (58.5%)	102 (75.0%)	0.022
Diarrhea	56 (87.5%)	124 (91.2%)	0.453
Mucous bloody stool	34 (50%)	101 (74.3%)	<0.001
Pure bloody stool	23 (33.8%)	29 (21.3%)	0.031
Tenesmus	21 (30.9%)	46 (33.8%)	0.753
Systemic manifestations			
Fever	13 (19.1%)	53 (39.0%)	0.007
Extraintestinal manifestations	16 (23.5%)	27 (19.9%)	0.587
Articular involvement	8 (11.8%)	11 (8.1%)	0.446
Peripheral arthritis	4 (57.1%)	5 (45.5%)	
Axial arthritis	2 (28.6%)	5 (54.5%)	
Mucocutaneous manifestations	2 (2.9%)	8 (5.9%)	0.501
Ocular lesions	0 (0.0%)	5 (3.7%)	0.172
Hepatobiliary diseases	13 (19.1%)	9 (6.6%)	0.009
PBC	1 (1.5%)	1 (0.7%)	1.000
PSC	2 (2.9%)	2 (1.5%)	0.602
Gallstones	5 (7.4%)	2 (1.5%)	0.036
Fatty liver disease	7 (19.3%)	3 (2.2%)	0.042
Thromboembolic diseases	3 (4.4%)	5 (2.5%)	0.336

PBC, primary biliary cholangitis; PSC, primary sclerosing cholangitis.

We did not detect any significant differences in complications or current infections between EO-UC and AO-UC patients, expect for severe lower gastrointestinal bleeding (0.0% *vs*. 8.1%, *p* = 0.017). There were no cases of toxic megacolon, intestinal perforation or intestinal obstruction in either group. The details are summarized in Table 3.

**Table 3:** Complications and infections.

Variable	EO-UC (*n* = 68)	AO-UC (*n* = 136)	*p*
Complications	7 (10.3%)	30 (22.1%)	0.053
Colonic stricture	5 (7.4%)	11 (8.1%)	1.000
Severe lower gastrointestinal bleeding	0 (0.0%)	11 (8.1%)	0.017
Perianal lesions	0 (0.0%)	7 (5.1%)	0.098
Intestinal intraepithelial neoplasia	1 (1.5%)	7 (5.1%)	0.273
UC-CRC	1 (1.5%)	4 (2.9%)	0.777
Infections	18 (26.5%)	28 (20.6%)	0.376
EBV infection	8 (11.8%)	12 (8.8%)	0.505
CMV infection	7 (10.3%)	14 (10.3%)	1.000
*Clostridioides difficile* infection	2 (2.9%)	3 (2.2%)	1.000
Fungal infection	6 (8.8%)	7 (5.1%)	0.365

UC-CRC, ulcerative colitis associated colorectal cancer; EBV, Epstein–Barr virus; CMV, cytomegalovirus.

Table 4 shows the laboratory and endoscopic evaluations of the UC patients. There were no significant differences between the two groups in terms of clinical classification, disease activity, or modified Mayo score. A significant difference in lesion location distribution was observed between the two groups (*p* = 0.001), with proctitis and left-sided colitis being more common in the EO-UC group than in the AO-UC group (14.7% *vs*. 2.2% and 22.1% *vs*. 18.4%, respectively).

**Table 4:** Laboratory and endoscopic evaluation.

Variable	EO-UC (*n* = 68)	AO-UC (*n* = 136)	Mean difference (95% CI)	*p*
Clinical classification				0.106
Initial-onset type	20 (29.4%)	25 (18.4%)		
Chronic relapsing type	48 (70.6%)	111 (81.6%)		
Lesion location				0.001
Proctitis	10 (14.7%)	3 (2.2%)		
Left-sided colitis	15 (22.1%)	25 (18.4%)		
Extensive colitis	43 (63.2%)	108 (79.4%)		
Disease activity				0.250
Remission phase	7 (10.3%)	6 (4.4%)		
Mild	12 (17.6%)	26 (19.1%)		
Moderate	30 (44.1%)	74 (54.4%)		
Severe	19 (27.9%)	30 (22.1%)		
Modified mayo score	7.99 ± 3.40	7.96 ± 2.99	0.029 (−0.89, 0.946)	0.950
Laboratory parameters
WBC	7.49 ± 2.79	7.72 ± 3.32	−0.06 (−1.12, 1.01)	0.620
HGB	116.62 ± 21.96	118.23 ± 28.54	−1.38 (−9.84, 7.07)	0.682
PLT	259.66 ± 92.95	309.89 ± 105.24	−43.97 (−78.41, −9.53)	0.001
Neutrophils%	65.94 ± 13.90	66.44 ± 11.84	−0.01 (−4.28, 4.26)	0.786
Albumin	36.25 ± 5.93	38.68 ± 6.89	−3.41 (−5.53, −1.28)	0.016
ESR	27.29 ± 22.34	22.84 ± 20.75	6.23 (−1.06, 13.51)	0.173
CRP	7.97 ± 19.51	5.06 ± 13.52	3.33 (−2.53, 9.18)	0.221
APTT	30.16 ± 7.81	32.41 ± 4.61	−2.68 (−4.98, −0.38)	0.012

WBC, white blood cell; HGB, hemoglobin; PLT, platelet; ESR, erythrocyte sedimentation rate; CRP, C-reactive protein; APTT, activated partial thromboplastin time.

We found that AO-UC patients and EO-UC patients exhibited comparable levels of circulating inflammation, with no differences in the white blood cells (WBCs), the percentage of neutrophils, hemoglobin (HGB), the erythrocyte sedimentation rate (ESR) or C-reactive protein (CRP) levels (*P* all > 0.05). Significant differences were observed in the platelet count (259.66 ± 92.95 *vs*. 309.89 ± 105.24, *p* = 0.001), serum albumin concentration (36.25 ± 5.93 *vs.* 38.68 ± 6.89, *p* = 0.016), and activated partial thromboplastin time (APTT) (30.16 ± 7.81 *vs*. 32.41 ± 4.61, *p* = 0.012).

### 3.2 Clinical management

**Figure 1:**
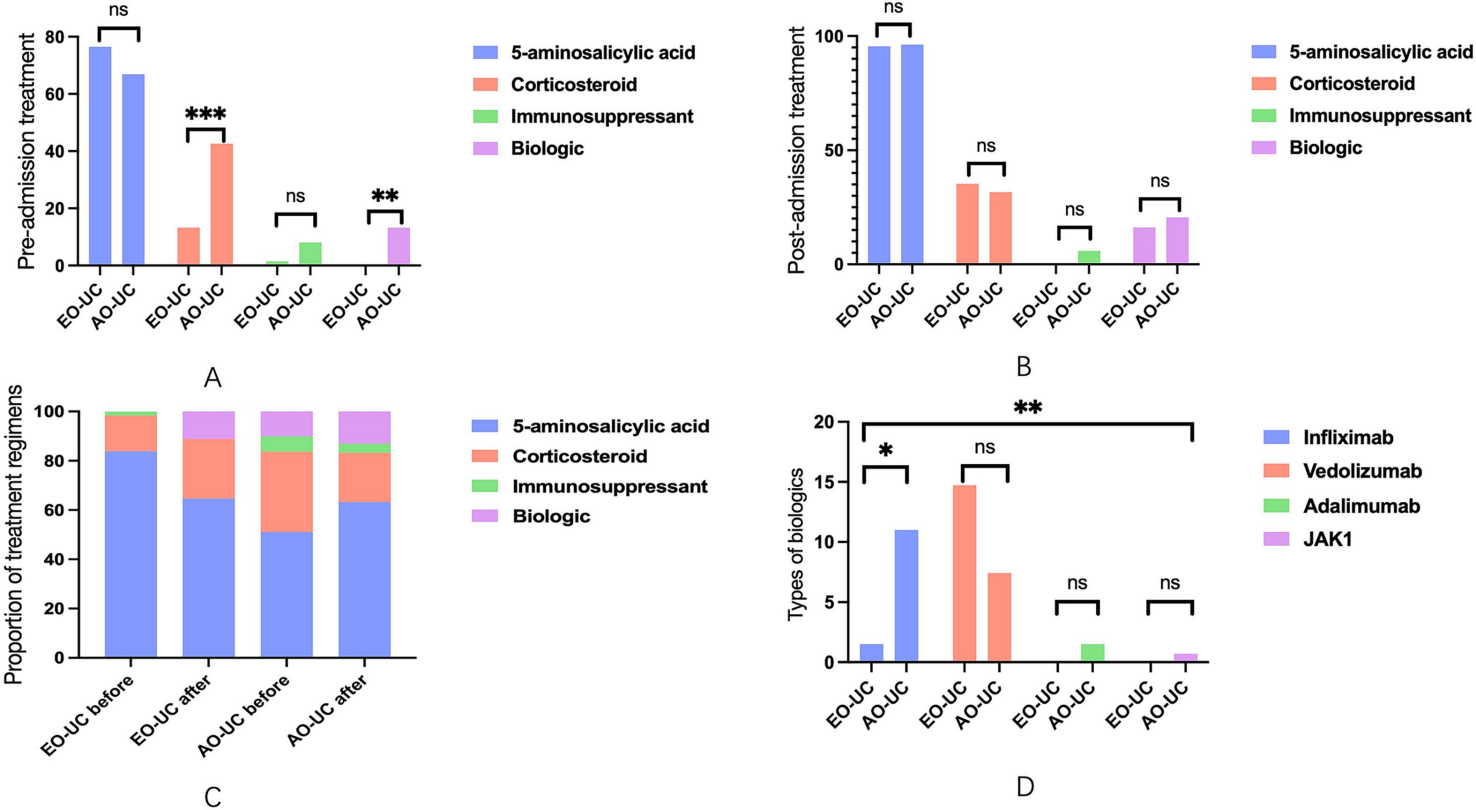
Comparison of treatment regimens between the EO-UC and AO-UC groups. **(A)** Differences in treatment regimens between the EO-UC and AO-UC groups before admission. **(B)** Differences in treatment regimens between the EO-UC and AO-UC groups after admission. **(C)** Stacked chart of treatment changes in patients in the EO-UC and AO-UC groups. **(D)** Differences between the two groups in the use of biological agents after hospitalization. *p < 0.05; **p < 0.01; ***p < 0.001; ns: not significant.

The adjustment of UC patients’ treatment before and after admission is shown in Figure 1. Prior to hospitalization, the EO-UC group demonstrated lower utilization rates of corticosteroids (13.2% *vs.* 42.6%, *p* < 0.001) and biologics (0.0% *vs.* 13.2%, *p* = 0.002) than the AO-UC group, whereas no significant difference was found in the immunosuppressant use (1.5% *vs.* 8.1%, *p* = 0.065) (Figure 1A). No statistically significant differences were observed in the adjustment of medication during hospitalization, as detailed in (Figure 1B). We compared the treatment regimens of the two groups before and after hospitalization (Figure 1C) and found that the proportions of EO-UC patients treated with corticosteroids (13.2% *vs*. 35.3%) and biologics (0.0% *vs*. 16.2%) increased after hospitalization. AO-UC patients were more likely to upgrade to biologics treatment (13.2% *vs*. 20.6%), especially infliximab (Figure 1D), and the usage rates of corticosteroids (42.6% *vs*. 6.9%) and immunosuppressants (8.1% *vs*. 5.9%) were reduced. Further analysis of the types of biologics used revealed that vedolizumab accounted for the greatest proportion of biologics used by EO-UC patients.

### 3.3 Clinical prognosis and outcomes

**Figure 2:**
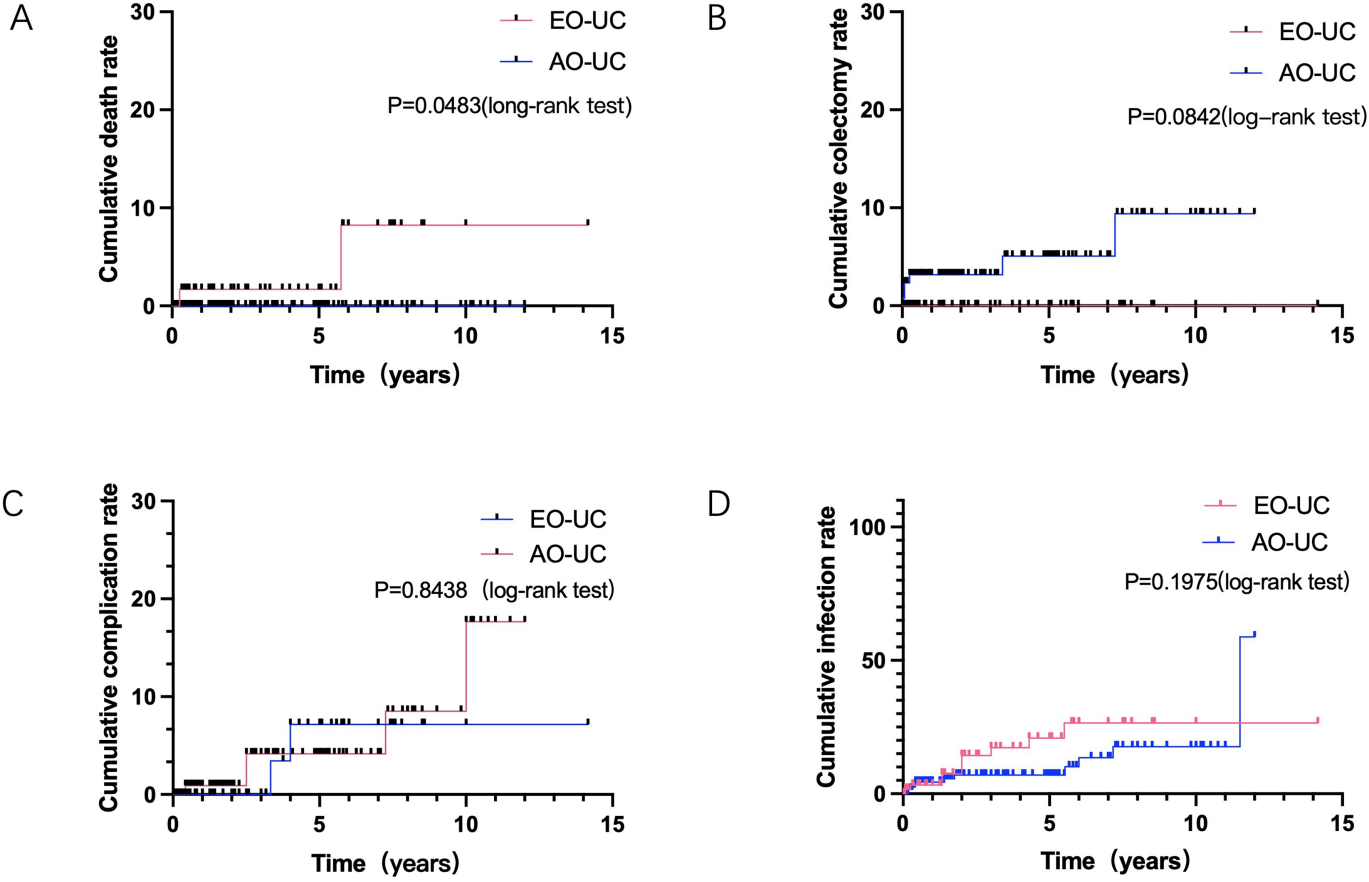
Kaplan–Meier curve of prognostic adverse events in patients with EO-UC and AO-UC. **(A)** Cumulative death rate in EO-UC vs. AO-UC. **(B)** Cumulative colectomy rate in EO-UC vs. AO-UC. **(C)** Cumulative complication rate in EO-UC vs. AO-UC. **(D)** Cumulative infection rate in EO-UC vs. AO-UC.

All patients were followed up until January 31, 2025 if available. Ultimately, we documented the follow-up information of 117 patients (66 patients/97.1% in the EO-UC group and 129 patients/92.6% in the AO-UC group), with no significant difference in the follow-up time (3.43 ± 2.95 years *vs.* 3.41 ± 3.32 years, *p* = 0.962). In addition, the mucosal healing time, relapse patterns and rehospitalization rate were comparable between the two groups. According to the results of the Kaplan–Meier analysis, as shown in Figure 2, a statistically significant difference in in-hospital all-cause mortality was observed between the EO-UC group and the AO-UC group (*p* = 0.0483), with a tendency toward higher in-hospital all-cause mortality in the EO-UC group, albeit based on a very small number of events. However, no statistically significant differences were found between the two groups in terms of UC-associated colectomies (*p* = 0.0842), complications (*p* = 0.8438) or infections (*p* = 0.1975).

**Figure 3:**
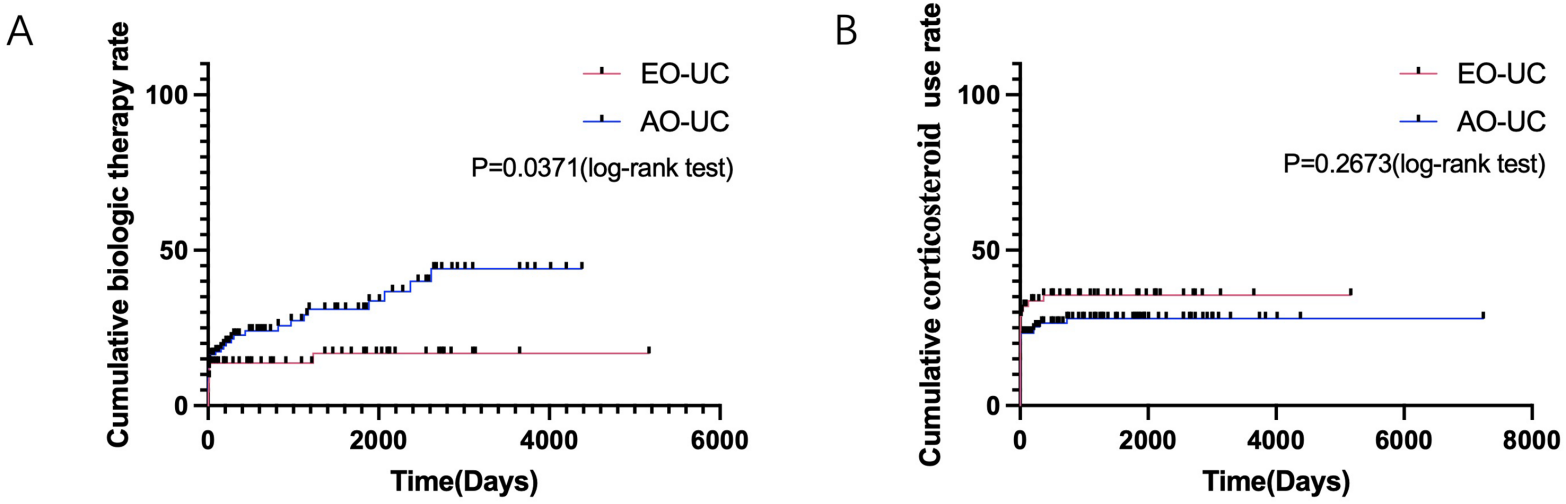
Kaplan–Meier curves of biologics and corticosteroid use in patients with EO-UC and AO-UC. **(A)** Cumulative biologic therapy rate in EO-UC vs. AO-UC. **(B)** Cumulative corticosteroid use rate in EO-UC vs. AO-UC.

Over the complete follow-up period, EO-UC patients showed higher 5-ASA utilization than AO-UC patients, with lower rates of immunosuppressant and biologic use. According to the results of the Kaplan–Meier analysis, as shown in Figure 3, there were significant differences in the cumulative biologic use rate (*p* = 0.0371). However, there was no significant difference in cumulative corticosteroid use between the two groups. The details are summarized in Table 5.

**Table 5:** Comparison of clinical prognosis.

Follow-up status	EO-UC (*n* = 66)	AO-UC (n = 129)	*p*
Follow-up duration (years)	3.43 ± 2.95	3.41 ± 3.32	0.962
Mucosal healing time (years)	2.28 ± 1.50	2.06 ± 2.35	0.834
Relapse pattern (follow-up >1 year)			0.699
Occasional	42 (84.0%)	72 (86.7%)	
Frequent	7 (14.0%)	8 (9.6%)	
Continuous pattern	1 (2.0%)	3 (3.6%)	
Rehospitalization due to relapse	17 (25.8%)	39 (30.2%)	0.513
Maintenance treatment options			0.084
5-ASA	50 (75.8%)	81 (62.8%)	
5-ASA+ immunosuppressants	1 (1.5%)	5 (3.9%)	
5-ASA+ biologics	6 (9.1%)	16 (12.4%)	
Immunosuppressants	0 (0.0%)	3 (2.3%)	
Biologics	4 (6.1%)	20 (15.5%)	
Biologics + immunosuppressants	2 (3.0%)	2 (1.6%)	
No medication	3 (4.5%)	1 (0.8%)	

5-ASA, 5-aminosalicylic acid.

## 4 Discussion

In this hospitalized cohort, we investigated the clinical manifestations, therapeutic strategies, and prognosis of UC patients, particularly those with EO-UC. We found that the EO-UC group was distinct from the AO-UC group in several characteristics, such as a higher proportion of males, higher rates of comorbidities and concomitant medication, milder clinical manifestations, and a greater proportion of rectum and left-sided colon involvement. 5-Aminosalicylic acid (5-ASA) remains the first-line therapeutic regimen. The EO-UC group had lower utilization rates of immunosuppressants and biologics; however, no significant differences were observed between the two groups in terms of clinical remission, relapse patterns, rehospitalization, complications, or infections. This finding is inconsistent with those of previous studies (12), which may be attributable to the fact that the two groups in our study had similar disease activity and similar remission induction regimens after admission. No adjusted model linking treatment exposure to clinical outcomes was constructed because of the limited number of events and because no independent association between treatment patterns and prognosis was demonstrated. Whether this difference contributes to differences in long-term prognosis remains unclear and warrants further investigation with larger sample sizes and multivariable adjustment.

A greater proportion of males was observed among EO-UC patients than among AO-UC patients, which aligns with previous findings (2, 4, 7). Elderly-onset patients displayed milder clinical manifestations, including lower frequencies of abdominal pain, mucoid bloody stools, and fever, than adult-onset patients did. These findings aligns with the observation that lesions in EO-UC patients are predominantly confined to the rectum and left colon. Moreover, this group showed a higher incidence of extraintestinal manifestations (19). Although the EO-UC group displayed milder clinical presentations, this study revealed no significant differences in disease severity between the EO-UC and AO-UC groups, which was consistent with the conclusions of a previous systematic review (7). Moreover, the EO-UC group had a greater comorbidity burden, which complicates clinical diagnosis and therapeutic management. The formulation of treatment regimens requires comprehensive consideration, posing challenges for clinicians in achieving timely induction of remission and maintaining this state.

Laboratory test results revealed that the albumin level was significantly lower in the EO-UC group than in the AO-UC group. This may reflect the poorer nutritional status of elderly patients. There was no significant difference in ESR or CRP levels, indicating that disease severity was comparable between the EO-UC and AO-UC groups. Previous studies have shown that PLT counts are higher in patients with UC than in healthy individuals and are positively associated with disease activity (20). In this study, platelet counts were lower in the EO-UC group, yet no significant difference in disease activity was observed between the two groups. This discrepancy may be attributed to the fact that, as an indirect marker of systemic inflammation influenced by multiple factors, platelet count does not exhibit a strict linear relationship with local intestinal inflammatory activity. UC patients were more likely to be hypercoagulable (21), and we found that the EO-UC group demonstrated a significantly shorter APTT than the AO-UC group, suggesting a potentially higher risk of thromboembolic events in this population. However, given the modest absolute difference and the exploratory nature of this analysis, these findings should be interpreted with caution.

Regarding initial treatment selection for EO-UC, the current findings reaffirm that 5-ASA remains the therapeutic cornerstone. This study revealed no significant differences in 5-ASA utilization rates between the two groups, although some patients required combination therapy with biologics or immunosuppressants to maintain remission. We found that 35.3% of EO-UC patients received corticosteroids during the induction of remission, which was consistent with findings reported by other scholars (22, 23). Kumar et al. revealed that there were no significant differences in corticosteroid utilization rates, dosage, administration routes, steroid resistance, or steroid dependence (24). We also observed similar results. In addition, the utilization rates of immunosuppressants and biologics were lower in elderly patients, which might be associated with higher risks of infection and malignancy (25, 26). Although azathioprine has been demonstrated to be effective and safe for maintaining remission in elderly patients with moderate-to-severe glucocorticoid-dependent UC, its use remains limited in EO-UC patients because of the increased incidence of adverse effects (27). This study revealed no statistically significant difference in the proportion of patients who received biologic therapy between the two groups, which aligns with findings from prior research (5). Studies indicate that biologics such as infliximab, vedolizumab, and ustekinumab demonstrate favorable efficacy and safety profiles in treating tEO-UC patients, suggesting substantial potential for broader application of biologics in EO-UC patient management (28, 29). This subgroup analysis revealed that the EO-UC group predominantly received vedolizumab. For nonresponsive patients, ustekinumab and upadacitinib were second-line solution choices, which may be attributable to their more favorable safety profiles (28, 30, 31). De Jong ME et al. demonstrated that elderly patients with IBD exhibited higher failure rates than younger patients when using anti-TNF therapy, along with increased incidences of serious adverse events and infections (29). Notably, compared with their adult-onset counterparts, patients with EO-UC have higher rates of anti-TNF agent discontinuation because of loss of response or adverse events (32, 33). Therefore, the application of anti-TNF agents is limited in EO-UC patients. Currently, vedolizumab and ustekinumab may be the preferred therapeutic agents for EO-UC patients. Although the clinical prospects of JAK inhibitors are promising, safety-related data remain limited (30).

Numerically higher risks of infection and in-hospital all-cause mortality were noted in the EO-UC group. Although elevated comorbidity burden in older adults may explain these results, the small event count prevented multivariable regression adjustment, and thus no causal relationships can be established (30, 34). Moroi et al. reported that in-hospital mortality rates were higher in the EO-UC group than in the AO-UC group, although no significant differences were found in therapeutic regimens or surgical rates (35, 36). The results of previous studies were not completely consistent, with some studies indicating a higher incidence of colorectal cancer (CRC) in EO-UC patients than in AO-UC patients (26), while a multicenter study revealed no difference in CRC incidence rates between the two groups (27, 37). No statistically significant differences were observed in UC-related CRC proportions in this study. A meta-analysis indicated that, compared with AO-UC patients, EO-UC patients had similar surgical risks at 1-year and 5-year intervals (38). Additionally, previous studies have shown that EO-UC patients have higher rates of UC-related surgeries (12, 14). This study further revealed that 6 cases of pancolectomy occurred in the AO-UC group, with surgical indications being extensive pancolonic inflammation refractory to medical therapy; however, no significant difference was observed between the two groups.

This study employed diagnostic age stratification, and summarized the data from two centers spanning 14 years. Our work provides a detailed analysis of clinical characteristics, therapeutic regimen selection, and prognosis in the EO-UC group, offering valuable insights for clinical management. However, this study has several limitations. First, the enrollment of hospitalized patients from two tertiary hospitals introduces potential selection bias, including possible referral bias. This cohort is likely skewed toward cases with more active and complex disease phenotypes and may not fully represent the overall spectrum of patients with UC. Consequently, our results may overestimate certain characteristics (e.g., comorbidity burden, medication use) and should not be directly generalized to non-hospitalized patients or those with mild disease managed in primary care. Second, the extended follow-up window may influence the analysis results because of evolving treatment strategies. Finally, as a retrospective study, several inherent limitations should be acknowledged. The number of hard clinical events was limited (only 2 deaths in the EO-UC group and 6 surgeries in the AO-UC group), which led to instability of the survival analyses and precluded a robust multivariable-adjusted analysis. Therefore, more systematic prospective studies with larger sample sizes are needed to further elucidate the clinical characteristics, prognosis, and pathogenesis of EO-UC patients in the future.

## 5 Conclusion

In conclusion, this study provides preliminary insights into the clinical characteristics and treatment patterns of elderly-onset versus adult-onset UC in a hospitalized cohort. In addition, EO-UC patients exhibited age-related complications, coagulation dysfunction, and concomitant medication use. Risks of infection, malignancy, and mortality in older adults may guide the selection of therapeutic regimens, which required comprehensive consideration. Finally, a better understanding of diagnostic age stratification may be used to facilitate personalized and effective treatment through timely risk–benefit assessments, enhance clinical monitoring of EO-UC patients, and proactively adjust treatment strategies on the basis of disease progression monitoring.

## Data Availability

The data analyzed in this study is subject to the following licenses/restrictions. The dataset contains confidential patient clinical data. Privacy and ethical restrictions prohibit public availability. Requests to access these datasets should be directed to correspondence author SD, e-mail: dingshigang222@163.com.

## Author’s note

The preliminary results of this study (abstract) were presented as a poster at the 21st Annual Congress of the European Crohn’s and Colitis Organisation (ECCO) and have been published as a conference abstract in the supplement of the Journal of Crohn’s and Colitis (39).
